# Can Thyroid Dysfunction Contribute to Infertility via Lipid Abnormalities?

**DOI:** 10.3390/cells15201847

**Published:** 2026-10-12

**Authors:** Rose B. Kelly, Aya A. Hassoun, Jan Stępniak, Piotr Kaczmarek, Małgorzata Karbownik-Lewińska

**Affiliations:** 1 Department of Endocrinology and Metabolic Diseases, Medical University of Łódź, 93-338 Łódź, Poland; rose.kelly@student.umed.lodz.pl (R.B.K.); aya.hassoun@student.umed.lodz.pl (A.A.H.); jan.stepniak@umed.lodz.pl (J.S.); piotr.kaczmarek@umed.lodz.pl (P.K.); 2 Polish Mother’s Memorial Hospital—Research Institute, 93-338 Łódź, Poland

**Keywords:** thyroid dysfunction, dyslipidemia, infertility, fecundability, cholesterol

## Abstract

**Highlights:**

**What are the main findings?**

- Thyroid dysfunction alters lipid metabolism, while adverse lipid profiles are associated with reduced fecundability and poorer outcomes in selected fertility-treatment studies.
- Experimental models suggest mechanisms through which lipid disturbances may impair reproductive cells, but mediation of thyroid-related infertility has not been demonstrated in humans.

**What are the implications of the main findings?**

- Thyroid evaluation and lipid reassessment after restoration of euthyroidism can help guide the management of dyslipidemia in reproductive-age adults.
- Further experimental studies are needed to test whether thyroid-related lipid changes contribute to reproductive dysfunction and clarify the underlying cellular mechanisms.

**Abstract:**

Thyroid dysfunction alters lipid metabolism and may impair reproduction, raising the possibility that lipid abnormalities contribute to thyroid-related infertility. This review evaluates dyslipidemia as a potential mechanistic link between these conditions. Thyroid hormones regulate hepatic LDL clearance, triglyceride-rich lipoprotein metabolism and HDL remodeling. Overt hypothyroidism commonly increases total and LDL cholesterol, changes that are partly reversible with levothyroxine, whereas lipid abnormalities in subclinical and central hypothyroidism are smaller and less consistent; hyperthyroidism generally lowers total and LDL cholesterol. Independently, adverse circulating lipid profiles are associated with reduced natural fecundability and poorer outcomes in selected assisted-reproduction studies. Experimental evidence identifies biologically coherent pathways involving ovarian steroidogenesis, follicular HDL function, oocyte cholesterol efflux and membrane signaling, endometrial receptivity and sperm membrane integrity. Human evidence supports both the thyroid–lipid and lipid–fertility relationships, but does not establish that lipid abnormalities mediate thyroid-related infertility. Interpretation is complicated by direct thyroid hormone effects, thyroid autoimmunity and metabolic confounding. Taken together, current findings support dyslipidemia as a biologically plausible potential contributor to thyroid-related reproductive dysfunction. Clinically, they suggest considering integrated assessment of thyroid and lipid status in reproductive-age adults and reassessment of the lipid profile after restoration of euthyroidism. Prospective preconception studies incorporating functional lipid measurements and formal mediation analyses are needed.

## 1. Introduction

Endocrine disorders are recognized secondary causes of dyslipidemia. The Endocrine Society lists hypothyroidism, acromegaly, Cushing syndrome and poorly controlled diabetes among the endocrine conditions that can alter the lipid profile, with hypothyroidism being the most common [1]. Secondary factors also include alcohol consumption and selected medications and account for a substantial proportion of dyslipidemia encountered in clinical practice [2]. Identifying thyroid dysfunction is, therefore, important when evaluating an abnormal lipid profile because the lipid disturbance may be partly reversible after restoration of euthyroidism.

Whereas a standard lipid panel reports total cholesterol (TC), low-density lipoprotein cholesterol (LDL-C), high-density lipoprotein cholesterol (HDL-C) and triglycerides (TGs), and these measurements are useful for characterizing systemic lipid metabolism, they provide limited information about lipoprotein composition and function. Serum concentrations may also differ from local lipid transport and cholesterol efflux within reproductive tissues, particularly within the ovarian follicle [3].

Thyroid hormones (THs), i.e., thyroxine (T4), also called a prohormone, and triiodothyronine (T3), being the active form, regulate lipid synthesis, mobilization and clearance in the liver and adipose tissue. Consequently, thyroid dysfunction can, therefore, increase or decrease concentrations of circulating lipid components depending on the direction and severity of the hormonal disturbance. Population-based evidence suggests that this relationship varies according to sex, age and menopausal status. For example, among 4275 Korean adults without previously diagnosed thyroid disease or treated dyslipidemia, the prevalence of biochemically defined thyroid dysfunction differed across TC, LDL-C and TG categories in women, particularly those younger than 55 years, but not in men or older women. No corresponding association was observed for HDL-C. In adjusted analyses among women, TSH was associated with elevated TC and LDL-C, whereas FT4 was inversely associated with hypertriglyceridemia [4]. Similarly, in the Thyroid Disorders, Iodine status and Diabetes Epidemiological (TIDE) survey in China, associations of overt and subclinical hypothyroidism with dyslipidemia were generally more apparent before than after menopause [5]. Although these cross-sectional findings do not establish direction or causality, they identify women of reproductive age as an especially relevant population in which to investigate thyroid–lipid interactions.

Because thyroid disorders may also affect reproductive function, thyroid-related changes in lipid metabolism provide a plausible link between the endocrine and reproductive systems. The central question of this review is whether dyslipidemia lies on the causal pathway from thyroid dysfunction to impaired fertility. Addressing it requires separating three related questions: whether thyroid dysfunction alters lipid metabolism, whether lipid abnormalities influence reproductive outcomes, and whether the lipid changes caused by thyroid dysfunction mediate impaired fertility. Evidence for the first two relationships does not by itself establish the third. This narrative review evaluates each link separately before considering their integration, potential confounding and alternative biological pathways.

## 2. Literature Search Strategy

Relevant publications were identified through targeted searches of PubMed/MEDLINE and by screening the reference lists of pertinent primary studies and reviews. Search terms combined concepts related to thyroid function, hypothyroidism, hyperthyroidism, dyslipidemia, cholesterol, lipoproteins, infertility, fecundability and assisted reproduction. Searches were updated through September 2026. Human observational, treatment and assisted-reproduction studies were considered alongside experimental work relevant to the proposed biological pathway. Because this was a narrative rather than a systematic review, studies were selected for relevance and methodological contribution, and no formal risk-of-bias assessment or quantitative evidence synthesis was undertaken.

## 3. Thyroid Hormones (THs) as Regulators of Lipid and Lipoprotein Metabolism

THs are important regulators of lipid homeostasis, acting predominantly through the liver but also through adipose tissue and peripheral pathways involved in lipoprotein turnover. In hepatocytes, T3 acts mainly through thyroid hormone receptor β (THRβ) and regulates the transcription of genes involved in fatty-acid and cholesterol metabolism. These transcriptional effects are complemented by changes in intracellular signaling, mitochondrial activity and autophagy [6,7,8].

Cholesterol is synthesized from acetyl-CoA through the mevalonate pathway, whose rate-limiting step is catalyzed by 3-hydroxy-3-methylglutaryl-coenzyme A reductase. Although THs stimulate this enzyme and may increase hepatic cholesterol synthesis, they simultaneously promote cholesterol clearance. T3 increases hepatic LDL receptor expression directly and through sterol regulatory element-binding protein 2, thereby enhancing the removal of LDL particles from the circulation [6,9]. Experimental studies also indicate that THs promote the conversion of cholesterol into bile acids and its biliary excretion. Thus, the net effect of thyroid hormone (TH) action depends on the balance between cholesterol synthesis, uptake and elimination rather than on changes in a single enzyme [6,8].

THs also regulate triglyceride-rich lipoproteins in the following way. T3 stimulates lipoprotein lipase, which hydrolyzes triglycerides carried in chylomicrons and very-low-density lipoproteins, and hepatic lipase, which participates in the remodeling of remnant and HDL particles. THs may additionally influence apolipoprotein A-V and LDL receptor-related protein 1, thereby contributing to triglyceride regulation and remnant clearance [6,9,10].

HDL metabolism is affected through the regulation of apolipoprotein A-I, scavenger receptor class B type 1, cholesteryl ester transfer protein and hepatic lipase. These pathways influence HDL formation, hepatic cholesterol uptake and particle remodeling. Reduced cholesteryl ester transfer protein and hepatic lipase activities in hypothyroidism may lead to the accumulation of larger HDL particles, whereas increased activity in hyperthyroidism may contribute to lower HDL-C. Nevertheless, changes in HDL-C are not uniform because THs influence several opposing stages of HDL metabolism. Furthermore, HDL function may be impaired despite normal or increased HDL-C concentrations [9,10].

Concerning fatty-acid metabolism, THs coordinate both synthesis and degradation. They stimulate hepatic lipogenesis but also promote lipid mobilization, lipophagy and mitochondrial β-oxidation. In adipose tissue, they influence lipolysis and the release of free fatty acids. When TH action is reduced, impaired fatty-acid oxidation and slower clearance of LDL and triglyceride-rich particles may predominate even though cholesterol synthesis is also decreased. This explains why hypothyroidism is generally associated with increased total and LDL cholesterol and with normal or elevated triglycerides, whereas hyperthyroidism usually produces lower total and LDL cholesterol concentrations [6,8,10].

**Figure 1:**
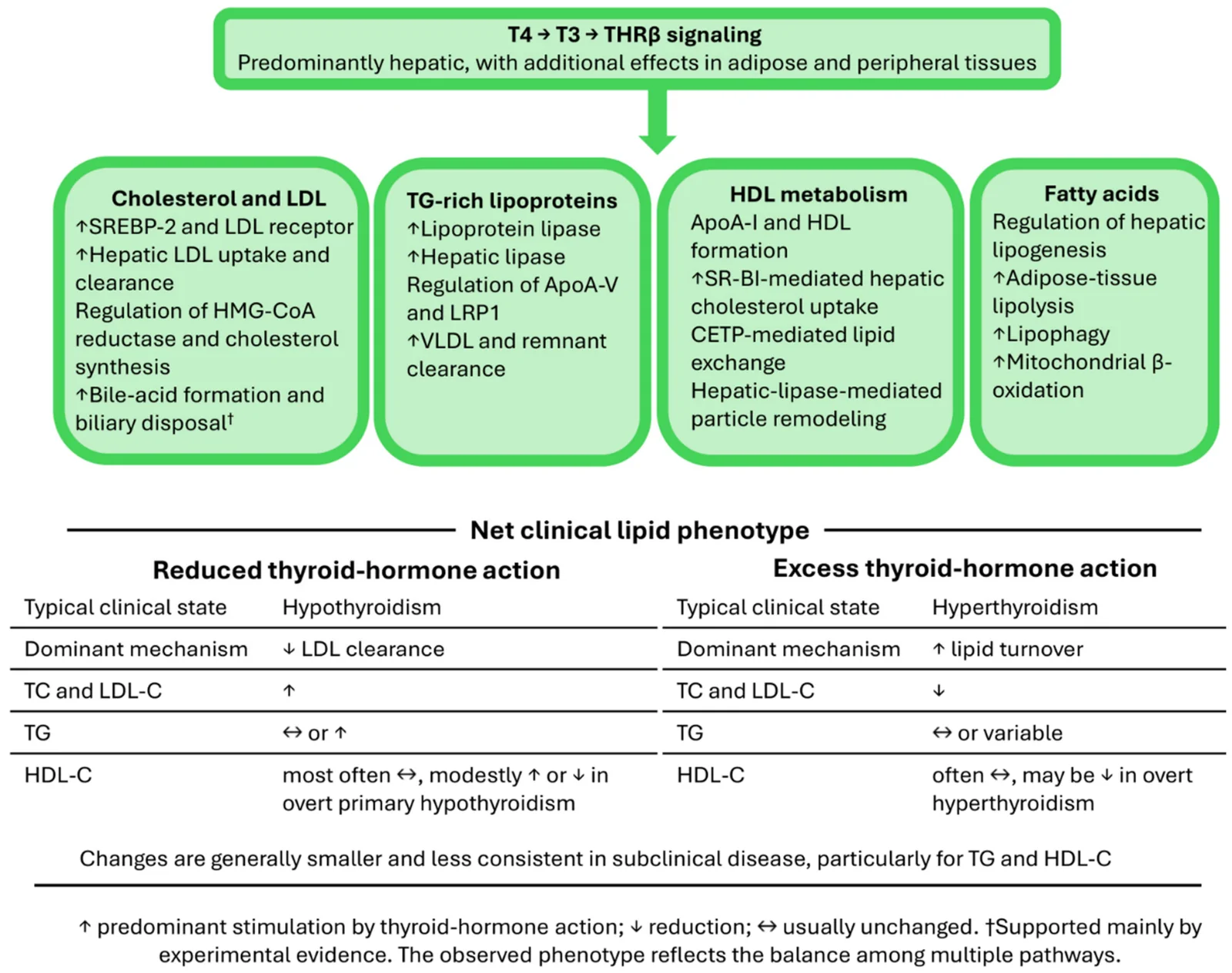
Principal pathways through which THs regulate lipid and lipoprotein metabolism. T3, acting predominantly through thyroid hormone receptor β in the liver, regulates LDL receptor-mediated clearance, cholesterol synthesis and elimination, the metabolism of triglyceride-rich lipoproteins, HDL remodeling and fatty-acid turnover. Reduced thyroid hormone action decreases LDL clearance and commonly increases total and LDL cholesterol, whereas excessive thyroid hormone action accelerates lipid turnover and usually lowers these fractions. The effects on triglycerides and HDL-C are more variable because they reflect several opposing regulatory processes. ApoA-I, apolipoprotein A-I; CETP, cholesteryl ester transfer protein; HDL, high-density lipoprotein; LDL, low-density lipoprotein; LRP1, LDL receptor-related protein 1; SR-BI, scavenger receptor class B type I; THRβ, thyroid hormone receptor β.

The principal pathways through which THs regulate lipid metabolism, together with the typical circulating lipid phenotypes of hypothyroidism and hyperthyroidism, are summarized in Figure 1.

## 4. Lipid Alterations Across Thyroid Dysfunction

### 4.1. Hypothyroidism

Hypothyroidism is a state of insufficient thyroid hormone action. It may be primary, owing to abnormalities at the level of the thyroid gland, or central, owing to inadequate stimulation by the pituitary or hypothalamus [11]. Primary hypothyroidism is classified biochemically as overt or subclinical. Serum TSH and free thyroxine (FT4) are the principal tests used to distinguish these forms, whereas central hypothyroidism requires interpretation of FT4 in the context of pituitary or hypothalamic disease because TSH may be low, normal or even mildly increased.

#### 4.1.1. Overt Primary Hypothyroidism

Overt primary hypothyroidism is defined by an increased TSH concentration together with a reduced FT4 concentration. Serum T3 is not required for the diagnosis and may remain within the reference range, particularly during earlier disease. Although reduced thyroid hormone action lowers HMG-CoA reductase activity, patients commonly have increased TC and LDL-C because LDL receptor expression and hepatic LDL clearance decrease to a greater extent [9,12].

TG concentrations may be normal or increased, partly because lower lipoprotein lipase activity delays the clearance of triglyceride-rich particles. HDL-C is often unchanged or modestly increased, particularly in the HDL_2_ fraction, when reduced hepatic lipase activity slows HDL_2_ catabolism. Changes in lipoprotein(a) have also been reported, although the findings are less consistent. The lipid phenotype is, therefore, dominated by increased TC and LDL-C rather than by a uniform change in every lipid fraction [9].

In the cross-sectional TIDE analysis, overt hypothyroidism was independently associated with hypertriglyceridemia (OR 1.50, 95% CI 1.12–2.01), and the association with elevated TG was observed in both premenopausal and postmenopausal women [5]. This finding supports an association between overt hypothyroidism and elevated TG but does not establish whether the lipid abnormality preceded the thyroid disorder or would improve following treatment.

Levothyroxine treatment provides clinical evidence that a substantial component of this dyslipidemia is thyroid-responsive. In a systematic review and meta-analysis, treatment of overt hypothyroidism reduced TC by approximately 58.4 mg/dL and LDL-C by 41.1 mg/dL on average [13]. More recent female-predominant prospective and retrospective cohorts reported directionally similar improvements in TC and LDL-C, with less consistent changes in TG and HDL-C [14,15]. Because these cohorts were uncontrolled and used heterogeneous treatment protocols, they reinforce treatment responsiveness but do not establish a dose-dependent lipid effect.

Overall, overt primary hypothyroidism is a reversible cause of secondary dyslipidemia. The evidence is strongest for increased TC and LDL-C and their reduction after restoration of euthyroidism, whereas effects on TG, HDL-C and lipoprotein(a) are more variable and depend on baseline metabolic characteristics and the completeness of thyroid hormone replacement.

Evidence specifically concerning women of reproductive age is limited but broadly consistent with the above pattern. In premenopausal women at low cardiovascular risk, overt hypothyroidism was associated with higher TC and LDL-C, whereas TG and HDL-C did not differ significantly from euthyroid controls; LDL-C correlated positively with TSH and inversely with FT4 [16]. Another case-control study found higher TC, LDL-C and TG and lower HDL-C in premenopausal women with newly diagnosed hypothyroidism, either subclinical or overt [17]. Short-term thyroidectomy-induced hypothyroidism was likewise accompanied by increased serum lipid concentrations in premenopausal women [18]. Collectively, these findings support the presence of thyroid-related lipid abnormalities at reproductive age, although small samples, heterogeneous definitions and residual metabolic confounding limit conclusions specific to this group of individuals.

#### 4.1.2. Subclinical Hypothyroidism

Subclinical hypothyroidism is defined by a persistently increased TSH concentration with FT4 remaining within the laboratory reference range. Its reported prevalence varies from approximately 3 to 15%, depending on age, sex, iodine status, the TSH threshold used and the population studied [19,20]. It is more common in women and in individuals with thyroid autoimmunity. Because mild TSH elevations may be transient, abnormal results should be confirmed before the condition is classified as persistent.

Compared with euthyroid controls, patients with subclinical hypothyroidism often have modestly higher TC and LDL-C, whereas findings for TG and HDL-C are less consistent [21,22]. A retrospective analysis of 2119 adults found higher TG, TC, LDL-C and very-low-density lipoprotein cholesterol in the subclinical hypothyroidism group [23]. However, the magnitude of lipid abnormalities varies with TSH elevation, age, adiposity, insulin resistance and coexisting diabetes.

Recent large studies illustrate this heterogeneity. In TIDE, subclinical hypothyroidism was associated with dyslipidemia among premenopausal women (OR 1.21, 95% CI 1.07–1.36), particularly with hypertriglyceridemia and low HDL-C, but not after menopause [5]. In a separate analysis of 55,303 Chinese adults aged 18–64 years, TSH of 4.21–6.57 mU/L was associated with modestly higher adjusted odds of dyslipidemia (OR 1.15, 95% CI 1.08–1.23), whereas a mild TSH elevation was not associated with the assessed metabolic disorders in adults aged 65 years or older [24].

By contrast, pooled individual-participant data from 69,006 adults in 16 cohorts found broadly similar conventional lipid concentrations in participants with subclinical hypothyroidism and euthyroidism in both women and men [25].

Levothyroxine treatment can produce modest reductions in TC and LDL-C in subclinical hypothyroidism, but the effect is smaller and more variable than in overt disease [13].

Studies focused on or predominantly including women of reproductive age further illustrate this variability. In premenopausal women with newly diagnosed mild subclinical hypothyroidism, TC and LDL-C were higher than in euthyroid controls and decreased after levothyroxine treatment [26]. In a 52-week double-blind, placebo-controlled trial involving patients with untreated subclinical hypothyroidism, most of whom were premenopausal women, levothyroxine reduced TC and LDL-C compared with placebo [27]. In a smaller study of premenopausal women with subclinical hypothyroidism, HDL-C was lower than in matched euthyroid controls and it increased following TSH normalization [28]. Among young women with polycystic ovary syndrome (PCOS), subclinical hypothyroidism has been associated with isolated increases in LDL-C or TG, although other analyses suggest that adiposity exerts a stronger influence on the overall metabolic profile than thyroid status [29,30,31]. These findings reinforce that neither the lipid phenotype nor the response to levothyroxine is uniform in reproductive-age women and that coexisting PCOS and adiposity must be considered.

#### 4.1.3. High-Normal TSH and Isolated Hypothyroxinemia

Biochemical variation within the euthyroid reference range has also been examined in women of reproductive age. A TSH concentration of at least 2.5 mIU/L that remains within the laboratory reference range should be described as high-normal TSH rather than subclinical hypothyroidism. In a retrospective analysis of 280 women with normal TSH, FT4 and FT3, abnormally high TG and an abnormally low HDL-C/TC ratio were more frequent among women with TSH of at least 2.5 mIU/L than among those with lower TSH values (17 versus 7% and 14 versus 5%, respectively). Thyroid peroxidase antibodies were also more frequent (27 versus 9%) [32].

Broader euthyroid cohorts have likewise associated higher TSH within the reference range with higher TC, TG or non-HDL-C and a less favorable cardiometabolic profile [33,34]. However, these studies were not restricted to women of reproductive age, and their observational designs do not establish that variation in TSH directly caused the lipid abnormalities.

In a prospective study of 99 euthyroid women of childbearing age, TSH of at least 2.5 mIU/L was associated with higher circulating lipid peroxidation (an index of oxidative damage to membrane lipids) products. Lipid peroxidation correlated positively with TG and inversely with HDL-C and the HDL-C/TC ratio [35]. A related study in the same population found lower concentrations of mannan-binding lectin (MBL)—a liver-derived protein involved in activation of the lectin pathway of complement—in women with high-normal TSH. Lower MBL was also associated with a less favorable HDL-C/TC ratio, suggesting a possible relationship between thyroid status, innate immunity and lipid metabolism [36].

These findings suggest that variation in thyroid status within the reference range may be associated with lipid and oxidative stress markers. Although the studies were small, they support the possibility that even subtle differences in thyroid function within the reference range may already be accompanied by measurable metabolic changes.

Isolated hypothyroxinemia, defined as reduced FT4 with a TSH concentration within the reference range, represents a distinct biochemical phenotype. In a retrospective analysis of 466 non-pregnant female endocrine inpatients, insulin resistance was found to be the only independent factor associated with isolated hypothyroxinemia. Across the larger analytical groups, rather than the eight affected women alone, FT4 correlated inversely with body mass index, LDL-C and TG and positively with HDL-C and the HDL-C/TC ratio [37]. These results associate lower FT4 with an adverse metabolic profile but do not establish a characteristic lipid phenotype or direction of causality for isolated hypothyroxinemia.

#### 4.1.4. Central Hypothyroidism

Central hypothyroidism results from pituitary or hypothalamic disease and is characterized by low FT4 with an inappropriately low, normal or even mildly increased TSH concentration. Unlike in primary hypothyroidism, TSH cannot reliably indicate disease severity or guide levothyroxine treatment; assessment should, therefore, rely primarily on FT4 and the clinical context [38].

In patients with multihormonal hypopituitarism, more advanced pituitary dysfunction and lower TSH have been associated with an unfavorable metabolic profile, including lower HDL-C [39]. However, lower TSH may reflect the severity of pituitary damage rather than thyroid hormone deficiency itself. Interpretation is further complicated by concomitant growth hormone, gonadal and adrenal deficiencies, their replacement therapies and differences in adiposity. In a Spanish cohort of 637 patients with central hypothyroidism, 34.2% had dyslipidemia at diagnosis, while 43.4% of those initially unaffected developed it during a median follow-up of seven years. Rates were similar in isolated and combined central hypothyroidism, although the small, isolated subgroup and retrospective design limit causal interpretation [40].

Treatment results suggest that inadequate thyroid hormone replacement may contribute to lipid abnormalities. In a randomized crossover study of 29 patients, body weight-adjusted levothyroxine targeting FT4 concentrations in the upper reference range reduced TC from 226 to 198 mg/dL and LDL-C from 135 to 116 mg/dL compared with a lower empirical dose; TG remained unchanged, while HDL-C decreased modestly [41]. Thus, adequate levothyroxine replacement may improve TC and LDL-C, but the independent contribution of central hypothyroidism to dyslipidemia remains difficult to separate from other features of hypopituitarism.

Evidence specifically addressing younger women remains limited and derives largely from studies of Sheehan syndrome. Mandal et al. [42] reported frequent dyslipidemia, particularly low HDL-C, although most participants had panhypopituitarism. Similarly, growth hormone-naïve women receiving conventional replacement therapy had higher TC, LDL-C and TG than matched controls [43]. These findings demonstrate an adverse lipid profile in relatively young women with central pituitary failure but cannot isolate the contribution of central hypothyroidism from concomitant growth hormone, gonadal and adrenal deficiencies or their treatment.

### 4.2. Hyperthyroidism

Hyperthyroidism is caused by excessive synthesis and secretion of THs and may be overt or subclinical. Graves’ disease, toxic multinodular goiter and toxic adenoma are common causes, while prevalence varies with iodine intake, age, sex, smoking and genetic susceptibility [44,45].

#### 4.2.1. Overt Hyperthyroidism

Overt hyperthyroidism is defined by a suppressed TSH concentration together with increased FT4 and/or T3. Increased thyroid hormone action produces a hypermetabolic state and frequently causes unintentional body mass loss. It also increases LDL receptor expression, cholesterol turnover and biliary cholesterol excretion. Consequently, TC and LDL-C are usually reduced, and HDL-C, apolipoprotein A-I, apolipoprotein B and lipoprotein(a) may also decline [9]. TG concentrations are often unchanged or variable. The resulting hypocholesterolemia reflects accelerated lipid turnover and should not automatically be interpreted as a uniformly favorable metabolic state.

Consistent with this expected pattern, the TIDE analysis associated overt hyperthyroidism with lower odds of hypercholesterolemia and high LDL-C overall and, of importance, among premenopausal women [5].

Restoration of euthyroidism reverses many of the above changes and is commonly followed by increases in TC, LDL-C, HDL-C and apolipoprotein B, whereas TG may remain unchanged [9,46].

Hyperthyroidism may also increase oxidative stress. For example, increased lipid peroxidation has been reported during levothyroxine overtreatment in patients after total thyroidectomy resulting in TSH suppression [47]. This pharmacologically induced subclinical hyperthyroid state provides indirect evidence that TSH suppression or relatively high thyroid hormone exposure may promote oxidative lipid damage.

Evidence specifically addressing women of reproductive age is limited but generally consistent with this pattern. In a prospective study of women aged 21–50 years with newly diagnosed Graves’ disease, restoration of euthyroidism with thioamides was accompanied by increases in TC, LDL-C and HDL-C [48]. Because body mass and fat mass also increased during treatment, the lipid changes cannot be attributed exclusively to normalization of thyroid hormone concentrations. In a smaller cross-sectional study, premenopausal women with overt hyperthyroidism had lower TC and higher markers of lipid and protein oxidation than euthyroid premenopausal controls [49]. These findings suggest that the characteristic low-cholesterol phenotype and increased oxidative stress may also be present at child-bearing age.

#### 4.2.2. Subclinical Hyperthyroidism

Subclinical hyperthyroidism is defined by a low or suppressed TSH concentration with FT4 and T3 remaining within their reference ranges [50]. Studies of its effects on the lipid profile are inconsistent. The TIDE analysis found no significant association of subclinical hyperthyroidism with dyslipidemia [5], while pooled data from 16 cohorts showed broadly comparable lipid concentrations except for a small reduction in LDL-C among women with subclinical hyperthyroidism [25]. Other studies describe small changes in the same direction as observed in overt hyperthyroidism [51,52]. Differences in the degree and duration of TSH suppression, disease cause, age and treatment probably contribute to this heterogeneity.

Taken together, thyroid status is an important determinant of systemic lipid metabolism. Evidence is strongest for overt primary hypothyroidism, in which TC and LDL-C commonly increase and improve after levothyroxine treatment. Lipid changes in the subclinical form, central hypothyroidism and borderline biochemical phenotypes are smaller or less consistent, whereas untreated hyperthyroidism usually lowers TC and LDL-C and lipid concentrations may rise after treatment. These observations establish a thyroid–lipid relationship but do not show that thyroid-related lipid changes account for impaired fertility. It should be stressed that published results do not suggest any specificity of lipid changes due to thyroid dysfunction in women of reproductive age. The following section, therefore, examines the separate clinical and mechanistic evidence linking lipid abnormalities with reproductive outcomes.

## 5. Lipid Profiles and Fertility

Lipids are indispensable for normal reproductive function. Cholesterol is a substrate for gonadal steroidogenesis and an essential structural component of cellular membranes, whereas fatty acids provide energy and serve as precursors of numerous signaling molecules. Reproductive competence, however, requires tightly regulated lipid synthesis, transport, storage and efflux. Consequently, both insufficient lipid availability and excessive lipid exposure or disturbed intracellular cholesterol trafficking may adversely affect fertility. Accordingly, assessment of the serum lipid profile, including TC, LDL-C, HDL-C and TG, provides a readily available clinical tool for identifying systemic disturbances in lipid metabolism that may be relevant to reproductive health. It can help characterize the metabolic milieu associated with reduced fecundability and adverse reproductive outcomes and facilitates comparisons across populations and clinical settings. However, it should be emphasized that these conventional measurements primarily quantify circulating lipid concentrations and provide limited information about lipoprotein characteristics and function. This distinction is particularly relevant to HDL because HDL-C quantifies the cholesterol carried by HDL particles rather than their functional properties. Moreover, differences between circulating and follicular-fluid HDL suggest that serum HDL-C may not fully represent local cholesterol homeostasis within the ovarian follicle [3].

### 5.1. Clinical Evidence Linking Dyslipidemia to Fertility and Reproductive Outcomes

Human studies have examined several related but distinct outcomes. Fecundability describes the probability of conception within a menstrual cycle and is commonly assessed through time to pregnancy, whereas infertility refers to failure to achieve pregnancy within a specified period of 12 months, with the precise threshold and diagnostic criteria varying among studies. Fertilization, embryo quality and live birth after assisted reproductive technologies (ARTs) represent treatment-specific outcomes, and early pregnancy loss concerns pregnancy maintenance rather than conception. These endpoints should not be treated as interchangeable, although together they can indicate where altered lipid metabolism may intersect with reproduction.

In the prospective Longitudinal Investigation of Fertility and the Environment (LIFE) study, 501 couples attempting pregnancy were followed for up to 12 months. Serum free, i.e., unesterified, cholesterol concentrations were higher among both female and male partners of couples who did not achieve pregnancy during follow-up. Higher female free cholesterol was associated with a longer time to pregnancy in both individual- and couple-based models, whereas the association for male free cholesterol was mainly evident when the lipid concentrations of both partners were considered simultaneously [53]. These findings suggest that disturbed cholesterol homeostasis in either partner may contribute to reduced couple fecundity. However, the study was observational and did not measure HDL-C or LDL-C; consequently, it neither established causality nor identified the lipoprotein fraction or reproductive pathway responsible for the association.

In the prospective Effects of Aspirin in Gestation and Reproduction (EAGeR) trial, preconception concentrations of TC, LDL-C, HDL-C and TG outside clinically desirable ranges were associated with reduced fecundability [54]. Several associations were attenuated after adjustment for body mass index or central adiposity, suggesting that obesity-related metabolic disturbances may partly explain the findings. Moreover, the cohort comprised women with one or two previous pregnancy losses rather than an unselected population attempting conception. The results, therefore, support an association between preconception lipid status and fecundability but do not clearly demonstrate that dyslipidemia is an independent cause of infertility.

Four cross-sectional analyses of partly overlapping NHANES cycles reported positive associations of self-reported infertility with the non-HDL-C/HDL-C ratio, Castelli risk index I, cardiometabolic index and triglyceride-glucose index [55,56,57,58]. Associations were generally modest, although women in the highest categories had approximately 1.7- to 2.2-fold higher adjusted odds of infertility than those in the lowest categories. Where reported, discrimination by an index alone was poor, with areas under the receiver operating characteristic curve of approximately 0.56–0.58.

These findings require cautious interpretation. The indices combine mathematically related lipid fractions or incorporate glucose and abdominal adiposity and, therefore, may reflect broader cardiometabolic dysfunction rather than a lipid-specific effect. The analyses also used partially overlapping populations, relied on self-reported infertility and measured current metabolic variables after the period during which infertility may have occurred. They should consequently be treated as related cross-sectional signals rather than four independent replications or evidence of temporal causality.

Mendelian randomization analyses provide evidence that is less susceptible to conventional confounding and reverse causation. Zhu et al. [59] reported that genetically predicted higher LDL-C and TC were associated with increased risk of female infertility in univariable analyses. After correlated lipid traits were considered simultaneously, LDL-C remained associated with infertility (OR 1.12, 95% CI 1.006–1.243). The modest effect size, possible genetic pleiotropy, restriction primarily to populations of European ancestry and heterogeneous etiologies included under female infertility nevertheless require cautious interpretation. These findings support a possible contribution of LDL-C but do not identify the reproductive pathway through which it might act.

Associations between circulating lipids and reproductive outcomes have also been reported in women undergoing ART. In a prospective cohort of women undergoing in vitro fertilization (IVF), higher serum TG concentrations around the time of oocyte retrieval were associated with a lower probability of live birth [60]. The relatively small sample and concurrent assessment of lipids and lipophilic micronutrients, however, limited conclusions regarding an independent effect of TG. In a real-world analysis of 5030 infertile women, dyslipidemia, particularly TC concentrations of at least 5.20 mmol/L, was negatively associated with live birth during the first complete IVF/intracytoplasmic sperm injection (ICSI) cycle [61]. Women with dyslipidemia more frequently displayed a type C endometrial pattern (which is generally considered less favorable for pregnancy outcomes compared to a traditional pattern), whereas the numbers of metaphase II oocytes and good-quality embryos did not differ substantially between groups. This observation does not establish impaired endometrial receptivity but suggests that the association with live birth may not be explained exclusively by poorer oocyte or embryo quality.

A further retrospective study found that dyslipidemia was negatively associated with the cumulative live-birth rate after IVF/ICSI in women without polycystic ovary syndrome (PCOS) [62]. Excluding PCOS reduced the influence of one important reproductive and metabolic confounder, although residual confounding by obesity, insulin resistance, diet and other metabolic abnormalities remained possible. Lipid status at the couple level was examined in a secondary analysis of 2370 couples seeking fertility treatment. Among couples planning IVF, elevated LDL-C in the male partner was associated with a lower probability of live birth. Moreover, the probability of live birth was lower when both partners had elevated TC or LDL-C than when both had concentrations within the normal range [63]. Comparable associations were not observed among couples planning non-IVF treatments. These findings indicate that assessment of female lipid concentrations alone may provide an incomplete picture of the relationship between dyslipidemia and couple fertility.

#### Early Pregnancy Maintenance

Evidence concerning pregnancy maintenance rather than conception comes from a retrospective single-center study of 1102 pregnant women. Higher TC and LDL-C measured at approximately 4 and 7 weeks of gestation were associated with early pregnancy loss, with the strongest estimates at 7 weeks (TC: OR 4.30, 95% CI 1.87–9.93; LDL-C: OR 5.17, 95% CI 2.14–12.49), whereas associations involving TG and HDL-C were less consistent [64]. Because lipid concentrations were measured after conception, these findings concern early pregnancy maintenance and cannot explain reduced fecundability.

Overall, human studies associate altered circulating lipid profiles and composite cardiometabolic indices with reduced natural fecundability, self-reported infertility, poorer selected ART outcomes and possibly impaired early pregnancy maintenance. However, the evidence is heterogeneous with respect to lipid exposure, study population and reproductive endpoint. Some studies assessed clinically defined dyslipidemia, whereas others examined lipid ratios, metabolic indices or differences within conventionally normal lipid ranges. Cross-sectional design, overlapping study populations, selection of women with previous reproductive problems, treatment-related heterogeneity and residual confounding by adiposity, insulin resistance, PCOS, diabetes, diet and lifestyle remain important limitations. The available findings, therefore, support an association but do not, by themselves, establish that circulating dyslipidemia is an independent cause of infertility.

### 5.2. Proposed Mechanisms Linking Dyslipidemia to Reproductive Dysfunction

Cholesterol has traditionally been considered essential for female fertility because it provides the substrate for ovarian steroidogenesis. Theca, granulosa and luteal cells obtain cholesterol through local synthesis, intracellular cholesteryl-ester stores and lipoprotein uptake, with receptor expression changing during follicular maturation and luteinization. Disturbed delivery could, therefore, alter estrogen or progesterone production. Nevertheless, reproductive function depends on maintaining cholesterol within a physiological range because both insufficient availability and excessive intracellular accumulation may be harmful [3].

Oocyte cholesterol homeostasis provides a more specific potential link between abnormal lipoprotein metabolism and impaired fertility. HDL is the predominant lipoprotein in follicular fluid, and its particle size, composition and associated functional components have been related to oocyte or embryo quality in women undergoing IVF [3,65]. Mammalian oocytes have limited capacity for *de novo* cholesterol synthesis and lack conventional HDL and LDL receptors; they, therefore, depend largely on cholesterol supplied by surrounding cumulus cells through gap junctions. Follicular-fluid HDL may help maintain homeostasis by accepting excess unesterified cholesterol from the oocyte. This function may be more relevant than serum HDL-C because HDL-C does not directly measure particle composition, antioxidant activity or cholesterol-efflux capacity.

Evidence for this pathway derives predominantly from genetically modified mice. Female mice lacking scavenger receptor class B type I develop abnormal unesterified-cholesterol-rich HDL, accumulate cholesterol in oocytes and are infertile despite preserved ovulation. Correction of the circulating lipoprotein abnormality restored fertility, indicating that the reproductive phenotype was not explained solely by inadequate ovarian steroidogenesis [66,67].

At the cellular level, ABCA1 and ABCG1 have been detected in mouse oocytes and appear to participate in cholesterol efflux to functional HDL [68]. Excess membrane cholesterol can modify lipid-raft organization and calcium signaling and reduce maturation-promoting factor and mitogen-activated protein kinase activity, thereby destabilizing metaphase II arrest and promoting inappropriate egg activation [3,69]. These experiments provide a coherent cellular mechanism, but they do not demonstrate that an equivalent pathway is driven by conventional serum dyslipidemia in women.

Although the results from animal studies cannot be directly extrapolated to humans, the above-described mechanisms should be considered, especially taking into account the protective nature of HDL molecules.

Lipid metabolism may also influence fertility at the level of the endometrium. Fatty acids and lipid-derived mediators, including prostaglandins, endocannabinoids, lysophosphatidic acid and sphingosine-1-phosphate, regulate embryo–endometrium communication, implantation, embryo spacing and decidualization [70]. Cholesterol-derived estradiol and progesterone coordinate the structural and functional transformation of the endometrium during the implantation window. Dyslipidemia-related lipotoxicity, oxidative stress, endothelial dysfunction and dysregulated inflammatory responses could, therefore, disturb the endometrial microenvironment. Excessive exposure to selected fatty acids may additionally affect cellular energy metabolism, endoplasmic-reticulum function and prostaglandin synthesis. Physiologically controlled inflammatory signaling is necessary for implantation; the proposed problem is inappropriate intensity or timing rather than inflammation itself. Evidence in humans remains indirect, and obesity, insulin resistance and PCOS may independently affect both lipid metabolism and endometrial function.

The association involving the TyG index (calculated from fasting TG and glucose concentrations) highlights insulin resistance as an overlapping pathway rather than evidence for a purely lipid-mediated mechanism. Insulin resistance may impair oocyte mitochondrial and glucose metabolism, reduce glucose uptake by granulosa cells, promote hyperandrogenism and contribute to endometrial inflammation, particularly in PCOS [58]. Because insulin resistance frequently coexists with hypertriglyceridemia, it may confound the lipid–fertility association or act as a parallel mediator. The TyG index cannot determine whether its triglyceride component has a direct reproductive effect.

Abnormal lipid metabolism may also compromise male fertility. Cholesterol is necessary for testicular steroidogenesis and contributes to sperm-membrane stability and fluidity. Controlled membrane remodeling and cholesterol efflux during epididymal maturation and capacitation are required for the acquisition of fertilizing ability and for the acrosome reaction. Spermatozoa are also rich in polyunsaturated fatty acids, which support membrane flexibility but make the cells highly susceptible to oxidative lipid damage. Lipid overload may, therefore, promote oxidative stress, membrane lipid peroxidation, mitochondrial dysfunction and abnormalities of sperm maturation, motility, capacitation and sperm–oocyte interaction [71,72]. In the male component of the LIFE study, higher serum TC, free cholesterol and phospholipid concentrations were associated with a lower percentage of spermatozoa with an intact acrosome and with altered sperm-head morphometry [71]. Associations with conventional semen parameters have not been consistent across human studies, and much of the mechanistic evidence remains animal-derived.

Taken together, the proposed mechanisms indicate that altered lipid homeostasis could affect ovarian steroidogenesis, oocyte competence, endometrial receptivity and sperm function. Direct human evidence is strongest for associations involving follicular-fluid composition and selected semen characteristics, whereas causal cellular evidence is derived mainly from animal or in vitro models. Insulin resistance, obesity and PCOS may intensify these processes or independently contribute to infertility.

**Figure 2:**
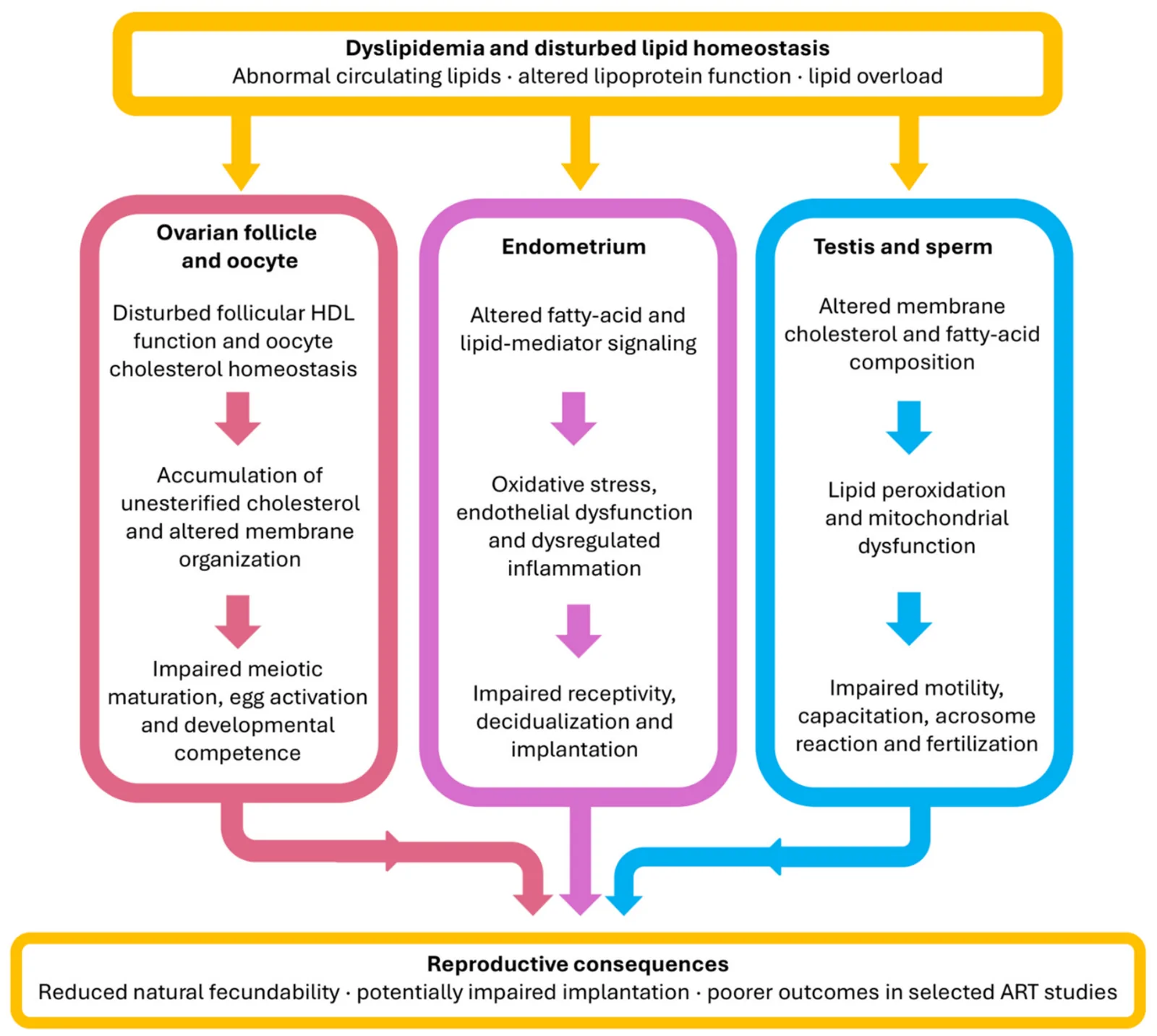
Proposed tissue-specific mechanisms linking dyslipidemia and disturbed lipid homeostasis to reproductive dysfunction. Dyslipidemia may affect oocyte competence, endometrial function and sperm function through distinct alterations in cholesterol handling, lipid-mediated signaling and oxidative damage. These pathways are supported to varying degrees, frequently by experimental evidence, while direct causal confirmation in humans remains limited. ART, assisted reproductive technology; HDL, high-density lipoprotein.

Proposed mechanisms linking dyslipidemia to reproductive dysfunction are presented in Figure 2.

## 6. Can Thyroid Dysfunction Contribute to Infertility Through Lipid Abnormalities?

The evidence supports two biologically connected but distinct relationships. First, reduced thyroid hormone action can impair hepatic LDL clearance and alter the metabolism of triglyceride-rich lipoproteins and HDL; this association is strongest in overt primary hypothyroidism and is partly reversible with levothyroxine [13,73]. Lipid changes are smaller and less consistent in subclinical or central hypothyroidism, while untreated hyperthyroidism generally lowers TC and LDL-C. Second, adverse circulating lipid profiles have been associated with reduced natural fecundability and selected ART outcomes. These component relationships are necessary for the proposed pathway but are not sufficient evidence of mediation.

A plausible cellular model applies most directly to hypothyroidism. Reduced T3 signaling through hepatic THRβ can lower LDL receptor-mediated clearance and disturb the remodeling of triglyceride-rich lipoproteins and HDL [6,10]. Resulting changes in lipoprotein composition or unesterified cholesterol could, in principle, alter follicular-fluid HDL and cholesterol flux among granulosa cells, cumulus cells and the oocyte. Impaired efflux could then modify oocyte membrane microdomains and calcium-dependent signaling, while dyslipidemia-related oxidative and inflammatory stress could affect the endometrium and sperm [68,69,70,72]. Each element has experimental support, but conventional serum TC, LDL-C, HDL-C and TG measurements do not unequivocally confirm that these local cellular events occur in women with thyroid dysfunction.

Human evidence obtained specifically in fertility settings remains indirect. In an exploratory analysis of 142 participants in the Environment and Reproductive Health Study who achieved pregnancy, higher preconception TT3 was associated with lower pregnancy HDL-C, while a higher FT3/FT4 ratio was associated with higher TC, non-HDL-C and LDL-C in the third trimester; TSH was not associated with the evaluated lipid parameters [74]. Because the analysis included only women who became pregnant and measured lipids after conception, it informs gestational lipid metabolism rather than whether thyroid-related dyslipidemia reduces the probability of conception.

Several alternative or overlapping pathways must also be considered. In euthyroid women undergoing IVF/ICSI, thyroid autoimmunity was associated with modestly poorer fertilization and cleavage-stage embryo measures but not with pregnancy, miscarriage or live birth; lipid variables were not measured [75]. This finding is compatible with immune or direct thyroid-related effects but provides no evidence of lipid mediation. Direct thyroid hormone effects on the hypothalamic–pituitary–gonadal axis and shared determinants such as obesity, insulin resistance and PCOS could likewise influence thyroid parameters, lipid metabolism and fertility independently.

Among the studies identified in this review, none simultaneously demonstrated that thyroid dysfunction preceded a reproductively relevant lipid alteration and that this alteration subsequently accounted for reduced fecundability or poorer ART outcomes. Establishing mediation will require prospective preconception measurements of thyroid function, thyroid antibodies, conventional and functional lipid variables, and reproductive outcomes in both partners. Studies conducted before and after restoration of euthyroidism should relate within-person lipid changes to time to pregnancy or ART outcomes and apply formal mediation analysis.

**Figure 3:**
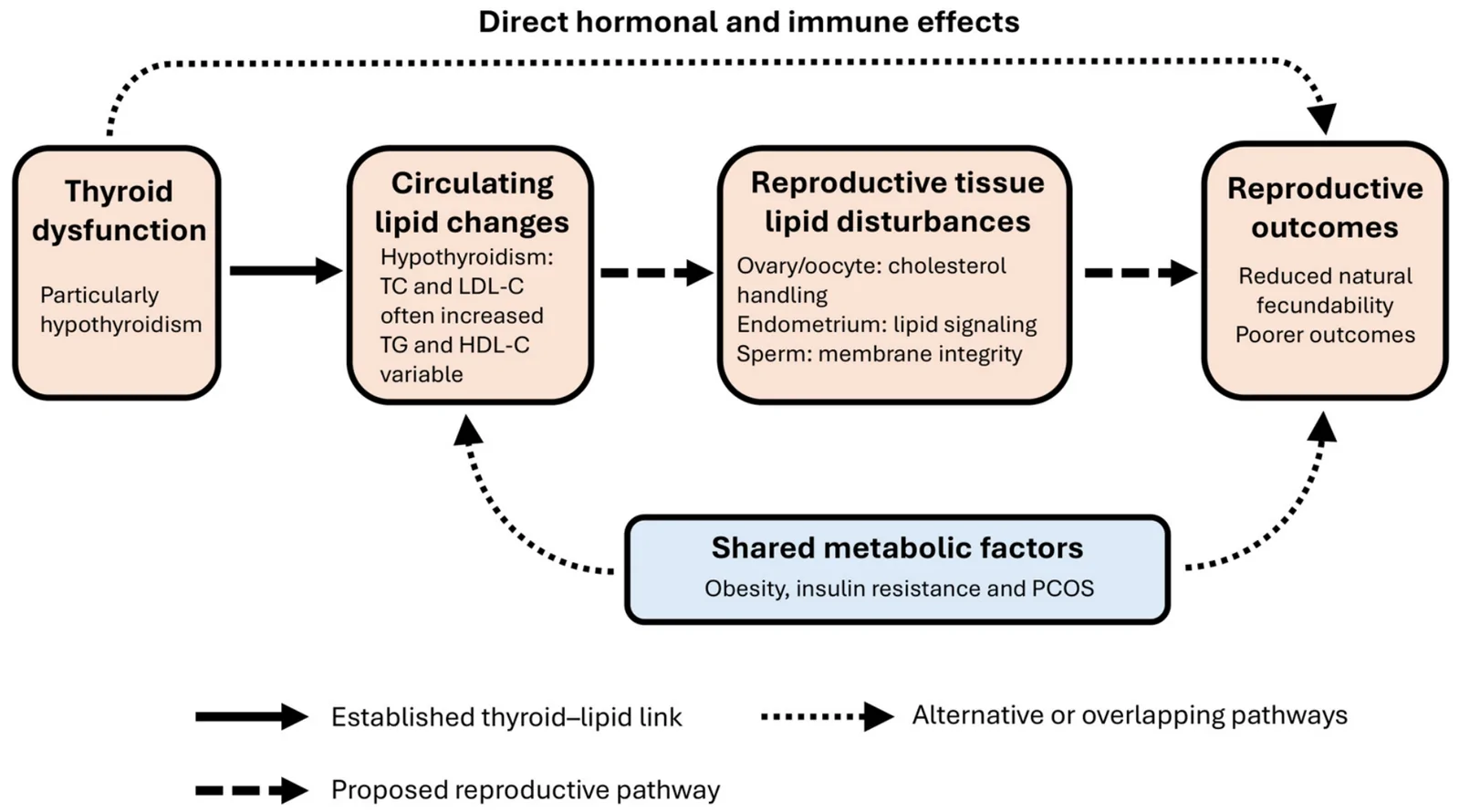
Proposed pathways linking thyroid dysfunction, lipid abnormalities and reproductive dysfunction. Thyroid dysfunction alters circulating lipid profiles, particularly in hypothyroidism. These changes may affect lipid homeostasis within reproductive tissues and contribute to impaired reproductive outcomes. Direct hormonal or immune effects and shared metabolic factors provide additional explanations. Solid arrows indicate the established thyroid–lipid relationship; dashed arrows indicate proposed pathways, including lipid mediation that remains to be demonstrated in humans.

What is the practical significance of all the above considerations? First, as recommended by some professional societies, such as the Polish Society of Endocrinology, all women of reproductive age planning pregnancy should be screened for thyroid dysfunction and appropriate treatment should be implemented when required, followed by careful monitoring [76,77,78]. Second, we suggest evaluating thyroid function in individuals of reproductive age of both sexes with lipid abnormalities, with thyroid-antibody testing when clinically indicated. This is consistent with lipid-management guidelines, which recommend excluding hypothyroidism as a cause of hyperlipidemia before initiating lipid-lowering treatment and reassessing the lipid profile after restoration of euthyroidism [1]. Third, we suggest considering appropriate treatment for dyslipidemia, when clinically indicated, in individuals of childbearing age with normal thyroid function. Fourth, as a research direction proposed by the authors, these clinical observations provide a rationale for further experimental studies in thyroid-cell models to clarify the cellular and molecular mechanisms underlying the interaction between thyroid function, lipid metabolism and reproductive effects. Proposed pathways linking thyroid dysfunction, lipid abnormalities and reproductive dysfunction are presented in Figure 3.

The main studies examining thyroid function, lipid homeostasis and reproductive outcomes are summarized in Table 1.

## 7. Conclusions

Thyroid dysfunction, particularly overt hypothyroidism, is an established and treatable cause of secondary dyslipidemia, while adverse lipid profiles are associated with reduced fecundability and selected reproductive outcomes. Experimental models identify biologically coherent pathways involving ovarian steroidogenesis, oocyte cholesterol efflux, membrane signaling, endometrial function and sperm integrity. Taken together, these findings support dyslipidemia as a biologically plausible potential contributor to thyroid-related reproductive dysfunction. However, human evidence does not establish that lipid abnormalities mediate the relationship between thyroid dysfunction and infertility. This proposed pathway provides a rationale for the integrated assessment of thyroid function, lipid status and fertility in prospective studies.

## Figures and Tables

**Table 1:** Summary of the main studies on thyroid function, lipid homeostasis and reproductive outcomes.

Study	Design and Population	Exposure or Intervention	Main Finding
**(A) Thyroid status and circulating lipid profiles**			
Oh et al. (2018) [4]	Cross-sectional KNHANES analysis; 4275 Korean adults without diagnosed thyroid disease or treated dyslipidemia.	Thyroid function and conventional lipid categories.	Associations involving TC, LDL-C and TG were observed mainly in women younger than 55 years; no corresponding association was found for HDL-C.
Han et al. (2022) [5]	Cross-sectional TIDE analysis; premenopausal and postmenopausal Chinese women.	Overt and subclinical thyroid dysfunction.	Subclinical hypothyroidism was associated with dyslipidemia before but not after menopause. Overt hypothyroidism was associated with higher TG; overt hyperthyroidism with lower odds of high TC and LDL-C.
Kotwal et al. (2020) [13]	Systematic review and meta-analysis of studies treating thyroid dysfunction.	Treatment of overt and subclinical hypothyroidism.	Treatment of overt hypothyroidism reduced TC by about 58.4 mg/dL and LDL-C by 41.1 mg/dL on average. Reductions were smaller and more variable in subclinical disease.
Torun et al. (2012) [16]	Comparative observational study; premenopausal women at low cardiovascular risk.	Overt hypothyroidism versus euthyroidism.	TC and LDL-C were higher in overt hypothyroidism; TG and HDL-C did not differ significantly. LDL-C correlated positively with TSH and inversely with FT4.
Baretella et al. (2025) [25]	Pooled individual-participant data; 69,006 adults from 16 cohorts.	Subclinical thyroid dysfunction versus euthyroidism.	Conventional lipid concentrations were broadly similar in subclinical hypothyroidism and euthyroidism. Women with subclinical hyperthyroidism had a small reduction in LDL-C.
Mikhail et al. (2008) [27]	52-week double-blind, placebo-controlled trial; untreated subclinical hypothyroidism, predominantly in premenopausal women.	Levothyroxine versus placebo.	Levothyroxine reduced TC and LDL-C compared with placebo.
Karbownik-Lewińska et al. (2020; 2017) [32,35]	Retrospective study of 280 women (2020) and a prospective study of 99 women (2017), all with normal thyroid tests.	High-normal TSH (≥2.5 mIU/L within the reference range).	Abnormally high TG and a low HDL-C/TC ratio were more frequent (2020); circulating lipid peroxidation products were higher (2017).
Slawik et al. (2007) [41]	Randomized crossover study; 29 patients with central hypothyroidism.	Weight-adjusted levothyroxine versus a lower empirical dose.	Targeting FT4 in the upper reference range reduced TC from 226 to 198 mg/dL and LDL-C from 135 to 116 mg/dL. TG was unchanged; HDL-C decreased modestly.
Chng et al. (2016) [48]	Prospective treatment study; women aged 21–50 years with newly diagnosed Graves’ disease.	Restoration of euthyroidism with thioamides.	TC, LDL-C and HDL-C increased as euthyroidism was restored.
**(B) Circulating lipids and reproductive outcomes**			
Schisterman et al. (2014) [53]	Prospective LIFE cohort; 501 couples attempting pregnancy, followed for up to 12 months.	Preconception serum unesterified cholesterol.	Higher female free cholesterol was associated with longer time to pregnancy. The male association was mainly evident in models including both partners.
Pugh et al. (2017) [54]	Prospective preconception lipid analysis within EAGeR; women with one or two previous pregnancy losses.	TC, LDL-C, HDL-C and TG outside desirable ranges.	Adverse preconception lipid profiles were associated with reduced fecundability; several associations weakened after adjustment for adiposity.
Zhang et al. (2025); Yang et al. (2025); Liu et al. (2025); Zhuang et al. (2024) [55,56,57,58]	Four cross-sectional analyses of partly overlapping NHANES populations of reproductive-age women.	NHHR, CRI-I, CMI and TyG index.	Higher indices were associated with self-reported infertility. Highest versus lowest categories showed about 1.7- to 2.2-fold higher adjusted odds; index-only discrimination was poor where reported.
Zhu et al. (2023) [59]	Univariable and multivariable Mendelian randomization; predominantly European-ancestry genetic data.	Genetically predicted circulating lipid traits.	Higher LDL-C and TC were associated with female infertility in univariable analyses. LDL-C remained associated in the multivariable model (OR 1.12, 95% CI 1.006–1.243).
Jamro et al. (2019) [60]	Prospective cohort of women undergoing IVF.	Serum TG around oocyte retrieval.	Higher TG was associated with a lower probability of live birth.
Yang et al. (2021) [61]	Real-world observational analysis; 5030 women undergoing a first complete IVF/ICSI cycle.	Dyslipidemia, particularly TC ≥ 5.20 mmol/L.	Dyslipidemia was associated with lower live-birth probability and more frequent type C endometrial patterns, without substantial differences in mature-oocyte or good-quality-embryo numbers.
Liu et al. (2021) [62]	Retrospective study; women without PCOS undergoing IVF/ICSI.	Dyslipidemia.	Dyslipidemia was associated with a lower cumulative live-birth rate.
Bollig et al. (2025) [63]	Secondary analysis; 2370 couples seeking fertility treatment.	Preconception lipid status in both partners.	Higher male LDL-C and elevated TC or LDL-C in both partners were associated with lower live-birth probability in couples planning IVF, but not non-IVF treatment.
Zhang et al. (2025) [64]	Retrospective single-center study; 1102 pregnant women.	TC and LDL-C at approximately 4 and 7 gestational weeks.	Higher TC and LDL-C were associated with early pregnancy loss, with stronger associations at 7 weeks. TG and HDL-C findings were less consistent.
**(C) Local mechanisms and evidence relevant to the integrated pathway**			
Kim et al. (2017) [65]	Human follicular-fluid study; women undergoing IVF.	Follicular-fluid HDL particle components.	HDL composition and associated components were related to embryo quality.
Trigatti et al. (1999); Miettinen et al. (2001) [66,67]	Genetically modified mouse models lacking SR-BI, including lipoprotein-correction experiments.	Disrupted HDL metabolism.	Female infertility occurred with abnormal unesterified-cholesterol-rich HDL. Correcting the lipoprotein abnormality restored fertility, arguing against steroidogenic insufficiency as the sole explanation.
Quiroz et al. (2020) [68]	Experimental study of mouse oocytes.	ABCA1/ABCG1 transporters and follicular-fluid HDL.	ABCA1/ABCG1 were detected in mouse oocytes and implicated in cholesterol efflux to HDL and regulation of oocyte cholesterol content.
Yesilaltay et al. (2014) [69]	Experimental mouse-egg study.	Excess oocyte cholesterol.	Excess cholesterol disturbed signaling and the maintenance of metaphase II arrest, promoting inappropriate egg activation.
Schisterman et al. (2014) [71]	Observational semen analysis within the male LIFE cohort.	Serum TC, free cholesterol and phospholipids.	Higher concentrations were associated with fewer spermatozoa with an intact acrosome and altered sperm-head morphometry.
Shen et al. (2025) [74]	Exploratory EARTH analysis; 142 fertility-center participants who achieved pregnancy.	Preconception thyroid function and gestational lipids.	Higher TT3 was associated with lower pregnancy HDL-C; a higher FT3/FT4 ratio with higher third-trimester TC, non-HDL-C and LDL-C. TSH was not associated with the assessed lipids.
Lu et al. (2025) [75]	Observational IVF/ICSI study; euthyroid women with or without thyroid autoimmunity.	Thyroid autoimmunity.	Autoimmunity was associated with modestly poorer fertilization and cleavage-stage embryo measures, but not pregnancy, miscarriage or live birth.

Studies are grouped by the relationship assessed. Observational associations, thyroid-treatment responses and experimental mechanisms represent distinct types of evidence. None of the included human studies establishes the complete thyroid–lipid–fertility mediation pathway. ABCA1/ABCG1, ATP-binding cassette transporters A1/G1; CI, confidence interval; CMI, cardiometabolic index; CRI-I, Castelli risk index I; EARTH, Environment and Reproductive Health Study; EAGeR, Effects of Aspirin in Gestation and Reproduction; FT3/FT4, free triiodothyronine/free thyroxine; HDL-C, high-density lipoprotein cholesterol; ICSI, intracytoplasmic sperm injection; IVF, in vitro fertilization; KNHANES, Korean National Health and Nutrition Examination Survey; LDL-C, low-density lipoprotein cholesterol; LIFE, Longitudinal Investigation of Fertility and the Environment; NHANES, National Health and Nutrition Examination Survey; NHHR, non-HDL-C/HDL-C ratio; OR, odds ratio; PCOS, polycystic ovary syndrome; SR-BI, scavenger receptor class B type I; TC, total cholesterol; TG, triglycerides; TIDE, Thyroid disorders, Iodine status and Diabetes Epidemiological survey; TSH, thyroid-stimulating hormone; TT3, total triiodothyronine; TyG, triglyceride-glucose index.

## Data Availability

No new data were created or analyzed in this review.
